# Risk Factors for Progression of Chronic Kidney Disease With Glomerular Etiology in Hospitalized Children

**DOI:** 10.3389/fped.2021.752717

**Published:** 2021-10-22

**Authors:** Guohua He, Chenglong Li, Xuhui Zhong, Fang Wang, Haibo Wang, Ying Shi, Lanxia Gan, Jie Ding

**Affiliations:** ^1^Department of Pediatrics, Peking University First Hospital, Beijing, China; ^2^Peking University Clinical Research Institute, Peking University First Hospital, Beijing, China; ^3^Clinical Trial Unit, The First Affiliated Hospital of Sun Yat-sen University, Guangzhou, China; ^4^China Standard Medical Information Research Center, Shenzhen, China

**Keywords:** chronic kidney disease, dialysis, progression, risk factor, hypertension

## Abstract

**Aim:** To Identify association between risk factors to Chronic kidney disease (CKD) stage 5 in children with glomerular diseases in children in China.

**Methods:** The Hospital Quality Monitoring System database was used to extract data for the study cohort. The primary outcome included progression to CKD stage 5 or dialysis. Cox regression was used to assess potential risk factors. Patients with lower stages (CKD stage 1 and 2) and higher stages (CKD stage 3 and 4) at baseline were analyzed separately.

**Results:** Of 819 patients (4,089 hospitalization records), 172 (21.0%) patients reached the primary outcome during a median followed-up of 11.4 months. In the lower stages group, factors associated with the primary outcome included older age [Hazard Ratio (HR), 1.21; 95% confidence interval (CI), 1.10–1.34] and out-of-pocket payment (HR, 4.14; 95% CI, 1.57–10.95). In the higher stages group, factors associated with the primary outcome included CKD stage 4 (HR, 2.31; 95% CI, 1.48–3.62) and hypertension (HR, 1.99; 95% CI, 1.29–3.07). The medical migration rate was 38.2% in this study population.

**Conclusion:** There are different risk factors for progression to the primary outcome in different stages in CKD with glomerular etiology. Further prospective studies are needed to assess these risk factors. The high medical migration rate reflected the regional disparities in the accessibility of pediatric kidney care between regions.

## Introduction

Chronic kidney disease (CKD) is a leading global health problem with a prevalence of 8–16% that continues to increase (1, 2). In recent years, increasing attention has been paid to childhood CKD. History of childhood kidney disease was found to be associated with a 3.19-times higher risk of end-stage kidney disease (ESKD) (3). Nearly 68% of children with CKD progress to ESKD by age 20 years (4). CKD with glomerular etiology was found to be associated with a significantly higher risk of CKD progression in children compared with non-glomerular etiology (5–9). Moreover, glomerular disease is the leading cause of CKD in India, Southeast Asia, and several other districts, with a prevalence ranging from 30 to almost 60% (10–17), and Asia has the largest CKD population (1, 18). Thus, it's important to identify risk factors for CKD progression in glomerular disease, especially in Asia.

Previous studies showed that advanced CKD stage, hypertension, anemia, older age, and female gender were related to a higher risk of CKD progression in children (5–8). However, these studies were performed in Europe and America pediatric CKD population, in which the dominant cause of CKD was non-glomerular disease such as congenital anomalies of the kidney and urinary tract (CAKUT) (5–8), and glomerular disease only made up <20% (5–8). Few studies had been concerned about the risk of progression in glomerular diseases in Asia children (19, 20). Additionally, children with CKD stage 1 were rarely included in previous studies because they were believed to have a low risk of progression. Consequently, little is known about the risk and risk factors of progression in stage 1.

China has a large CKD population (1, 2, 21), therefore, research on risk factors for the progression of CKD in Chinese children is helpful to reveal the potential risk factors for children CKD progression in Asia. We, therefore, conducted a cohort study based on Hospital Quality Monitoring System (HQMS), a national inpatient database in China, to evaluate the association between risk factors and progression to CKD stage 5 in CKD children with glomerular etiology.

## Methods

### Patients Characteristics

HQMS database is the largest national inpatient database in China, authorized by the National Health Commission of the People's Republic of China. There are over 190 million standardized inpatient records from 998 tertiary hospitals across 31 provinces in China in the HQMS database. There are up to 353 variables including patient demographics, clinical diagnosis coded according to the International Classification of Disease, 10th revision (ICD-10), medical payment methods, and information on hospitals on every record. Owing to the big data stored in the HQMS, numerous studies have been published on CKD (21–23), cardiovascular diseases (24), and other diseases (25).

Records of patients under 18 years old who were admitted between June 1, 2013, and December 31, 2018, were screened. Patients who were hospitalized more than once with a diagnosis of any stage from CKD stage 1 to stage 5 or dialysis were identified by ICD-10 disease codes. Patients with missing information on sex were excluded because sex was considered a primary parameter for evaluating the quality and completeness of records. Patients with the diagnosis of CKD stage 5 (including the diagnosis of chronic kidney failure) or dialysis on the first admitted record were excluded because these diagnoses were defined as the outcome. The details of patients excluded for this group were shown in Supplementary Data 1. Patients with the primary cause of non-glomerular diseases or unknown causes of CKD were excluded. The details of non-glomerular diseases excluded were shown in Supplementary Data 2. The study protocol was reviewed and approved by the Ethics Committee Board of Peking University First Hospital [Approval Number: 2021 (009)].

### Data Collection

Extensive demographic and clinical data were extracted from each record, including age, sex, admission date, location of residence, location of the hospital, payment method and the hospitalization counts for each patient during study period. Stage of CKD, specific disease as the cause of CKD, comorbidity with hypertension, and comorbidity with anemia was acquired from the recorded diagnoses (including one primary diagnosis and up to nine other diagnoses). For diagnosis of hypertension, HQMS use the measurement of BP more than 95th in the same sex and age group at 3 different visits in clinical practice according to the 2010 Chinese guidelines for the management of hypertension (26), which is consistence with the pediatric hypertension recommendations in Europe (27) and United States (28). Stages of CKD were extracted from each discharge record as a time-dependent parameter. Diagnoses in all records of the same individual were analyzed to obtain the specific disease as the cause of CKD. For comorbidity classification, patients were classified as hypertension vs. non-hypertension and anemia vs. non-anemic, according to their diagnosis. Payment methods were classified as out-of-pocket or basic medical insurance according to the patient's payment information in each record. The location of residence (province-level) of each patient was identified from the address, hometown, and zip code on each record. The location of the hospital (province-level) was identified from the address of the hospital. Medical migration (province-level) was defined as a patient left their location of residence and traveled to a hospital in another province for admission. Followed-up time was calculated from the admission date of the first record to the admission date of the last record of the same person.

### Study Outcomes

The primary outcome was progression to CKD stage 5, which was defined as a diagnosis of CKD stage 5 (including the diagnosis of chronic kidney failure), or dialysis (including peritoneal dialysis and hemodialysis) in the HQMS records. These diagnoses were identified by ICD-10 accordingly. The followed-up time was calculated from the admission date of the first record to the admission date of the last record of the same patient.

### Statistical Analysis

Patients were classified into lower stages group and higher stages group according to their first discharge record. Baseline features of different stages groups were compared using chi-squared tests for categorical data and Wilcoxon rank-sum tests for non-Gaussian continuous data.

For those who reached the primary outcome, the time-to-event was calculated from the admission date of the first record during the study period to the admission date of record when the primary outcome was reached. For those without a primary outcome in the study period, the time-to-event was calculated from the admission date of the first record to the admission date of the last record.

Cox proportional hazard models were used for multivariate analysis to evaluate independent risk factors for the primary outcome, with missing observations excluded. Variables examined in the multivariate analysis included age, sex, hypertension, anemia, medical migration, and payment methods. For analysis purposes, specific disease as the cause of CKD was classified into four groups: nephrotic syndrome, lupus nephritis, IgA nephropathy, and miscellaneous disease. Given the heterogeneity in baseline characters and interaction between CKD stages and other factors, patients in the lower stages group and higher stages group were analyzed separately. A likelihood ratio-based test was performed to test the null hypothesis that the coefficient in the regression equaled zero, with a two-tailed alpha of 0.05 considered statistically significant. Covariates within the pre-defined statistical significance level were retained in the final model. Hazard ratios (HRs) were used to measure the association with progression. The proportional hazard assumption was checked using weighted Schoenfeld residuals, with violated covariates included as time-dependent covariates.

All analyses were performed using SAS software 9.4 (SAS Inc., Cary, NC, USA), with confidence level of 95% used for interval estimates, unless otherwise specified.

## Results

### Study Population

**Figure 1:**
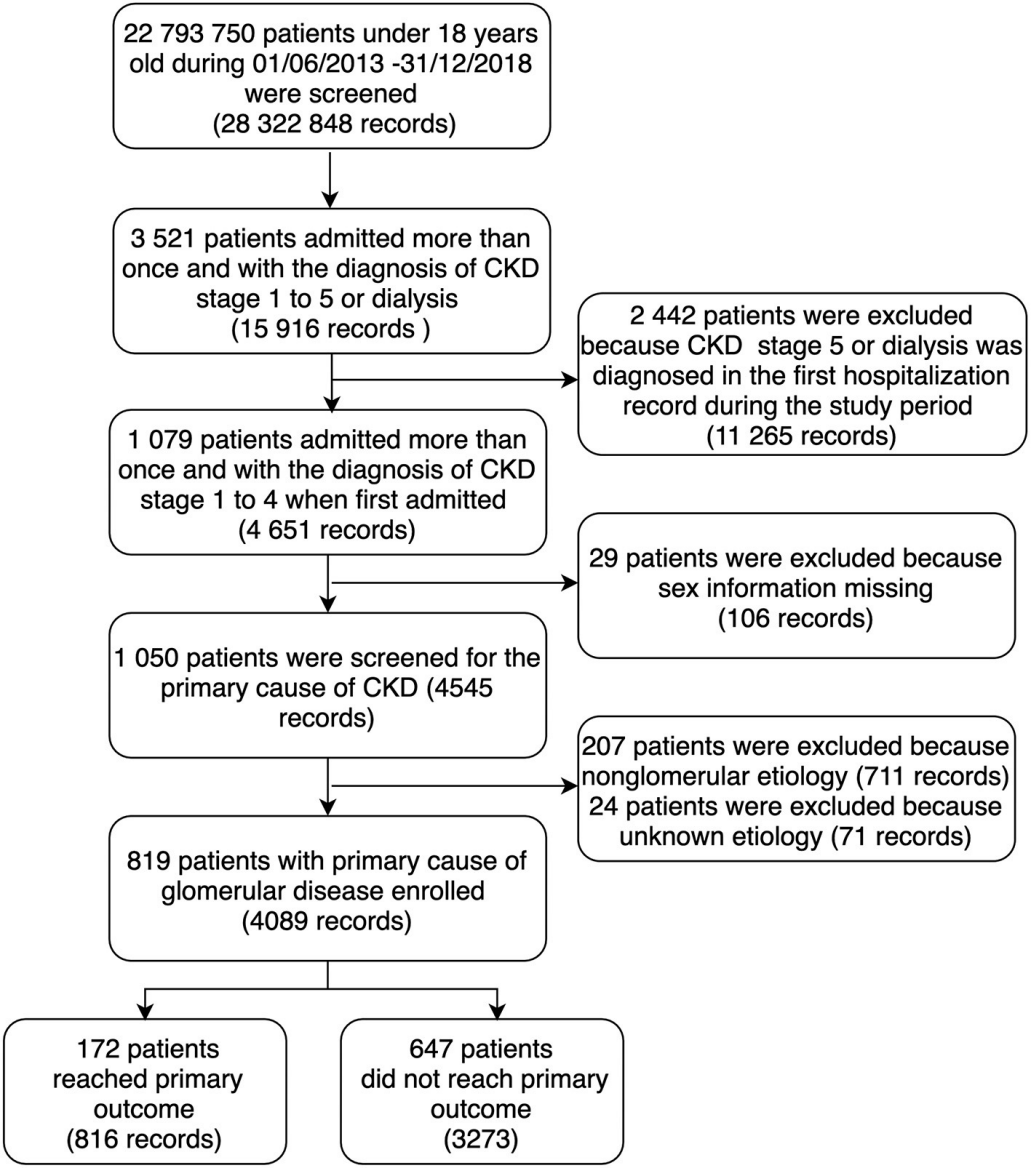
The procedure of study population selection.

There were 819 patients (4,089 hospitalization records) included in the final analysis. The study population selection process was shown in Figure 1. Demographic and clinical data pertaining to included patients are shown in Table 1. Among the 819 patients, 498 (60.8%) were boys, and the median age at enrollment was 11.9 [interquartile range (IQR), 7.9–15.6] years old. There were 549 (67.0%) patients with CKD stage 1, 59 (8.4%) with CKD stage 2, 127 (15.5%) with CKD stage 3 and 84 (10.3%) with CKD stage 4 at first admitted. The three most common specific disease causes were nephrotic syndrome (53.8%), lupus nephritis (16.7%), and immunoglobulin A (IgA) nephropathy (10.7%). Details on disease are shown in Supplementary Data 3. The prevalence of hypertension was 27.0% (148/549) in CKD stage 1, 33.9% (20/59) in stage 2, 48.8% (62/127) in stage 3, and 53.6% (46/84) in stage 4. Out-of-pocket payment made up 67.2% of the payment methods in the study population.

**Table 1:** Patient characteristics in lower stages group and higher stages group.

**Characteristics**	**Lower stages group *N* = 608**	**Higher stages group *N* = 211**	***P*-value**
Male sex, *N* (%)	374 (61.5)	124 (58.8)	0.4815
Age, years	11.0 (6.8, 14.7)	13.9 (11.4, 16.5)	<0.0001
Stages of CKD, *N* (%)			<0.0001
CKD 1	549 (90.3)	NA	
CKD 2	59 (9.7)	NA	
CKD 3	NA	127 (60.2)	
CKD 4	NA	84 (39.8)	
Disease, *N* (%)			0.0014
Nephrotic syndrome	350 (57.6)	91 (43.1)	
Lupus nephritis	97 (16.0)	40 (19.0)	
IgA nephropathy	63 (10.4)	25 (11.8)	
Miscellaneous diseases^†^	98 (16.1)	55 (26.1)	
Hypertension, *N* (%)	168 (27.6)	107 (50.7)	<0.0001
Anemia, *N* (%)	207 (34)	128 (60.7)	<0.0001
Payment methods, *N* (%)			0.0001
Out-of-pocket	439 (72.2)	111 (52.6)	
Basic medical insurance	155 (25.5)	77 (36.5)	
Unknown	14 (2.3)	23 (10.9)	
Total number of hospitalization, *N*	3,282	807	
Medical migration, *N* (%)			0.4840
Yes	228 (37.5)	85 (40.3)	
No	376 (61.8)	125 (59.2)	
Unknown	4 (0.7)	1 (0.5)	
Followed-up time, months	11.4 (4.5, 24.9)	12.1 (4.8, 24.3)	0.8252
Reach primary outcome, *N* (%)	43 (7.1)	129 (61.1)	<0.0001

*Values for categorical variables are given as number (percentage); value for age as median (Interquartile range)*.

† *The group of “Miscellaneous diseases” with 153 patients included 78 with Henoch-Schönlein purpura nephritis, 24 with Alport syndrome, 22 with ANCA glomerulonephritis 12 with membrane nephropathy, and 17 with other known diagnoses*.

*NA, Not applicable*.

During a followed-up of 11.4 (IQR, 4.6–21.4) months, 172 (21.0%) patients reached the primary outcome including 43 (7.1%) patients in the lower stages group and 129 (61.1%) patients in the higher stages group. Patient characters classified by the outcome were shown in Table 2 and Supplementary Data 4, 5. Among the progressors (*n* = 43) in lower stages group, no statically significant difference was observed in the numbers of hospitalization between out-of-pocket payment patients than those paid by insurance [33 patients with out-of-pocket payment, total number of hospitalization = 203, median 4.0 (IQR, 2.0–9.0) vs. 8 patients with insurance, total number of hospitalization = 49, median 4.0 (IQR, 3.0–7.0), *p*-value for difference = 0.8542. There were 2 patients whose payment method unknown].

**Table 2:** Baseline characteristics of 172 patients in progress group classified by CKD stages.

**Characteristics**	**CKD 1** ***N* = 26**	**CKD 2** ***N* = 17**	**CKD 3** ***N* = 67**	**CKD 4** ***N* = 62**
Male sex, *N* (%)	15 (57.7)	10 (58.8)	46 (68.7)	34 (54.8)
Age, years	12.2 (9.5, 16.0)	16.0 (13.9, 17.5)	14.0 (11.2, 16.3)	14.3 (11.7, 16.9)
Disease, *N* (%)				
Nephrotic syndrome	11 (42.3)	9 (52.9)	31 (46.3)	33 (53.2)
Lupus nephritis	8 (30.8)	5 (29.4)	11 (16.4)	4 (6.5)
IgA nephropathy	3 (11.5)	1 (5.9)	8 (11.9)	7 (11.3)
Miscellaneous diseases	4 (15.4)	2 (11.8)	17 (25.4)	18 (29.0)
Hypertension, *N* (%)	13 (50.0)	9 (52.9)	39 (58.2)	37 (59.7)
Anemia, *N* (%)	12 (46.2)	9 (52.9)	36 (53.7)	43 (69.4)
Payment methods, *N* (%)				
Out-of-pocket	20 (76.9)	13 (76.5)	33 (49.3)	34 (54.8)
Basic medical insurance	6 (23.1)	2 (11.8)	27 (40.3)	21 (33.9)
Unknown	0 (0.0)	2 (11.8)	7 (10.4)	7 (11.3)
Total number of hospitalization, *N*	3,038	244	493	314
Medical migration, *N* (%)				
Yes	9 (34.6)	3 (17.6)	31 (46.3)	16 (25.8)
No	17 (65.4)	14 (82.4)	35 (52.2)	46 (74.2)
Unknown	0 (0.0)	0 (0.0)	1 (1.5)	0 (0.0)
Progressors, *N*/Total number in this stage, *N* (%)	26/549 (4.7)	17/59 (29.8)	67/127 (52.8)	62/84 (73.8)

*Values for categorical variable are given as number (percentage); value for age, and hospitalization count, as median (Interquartile range)*.

### Multivariate Analysis for Progression

Multivariate proportional hazard regression analyses were used to evaluate independent risk factors for the primary outcome. The results are shown in Table 3. There were 608 patients in the lower stages group. In this group, older age (HR, 1.21; 95% CI, 1.10–1.34), and out-of-pocket payment method (HR, 4.14; 95% CI, 1.57–10.95) were significantly associated with a higher risk of the primary outcome. There were 211 patients in the higher stages group. CKD stage 4 (HR, 2.31; 95% CI, 1.48–3.62) and hypertension (HR, 1.99; 95% CI, 1.29–3.07) were significantly associated with higher risks of the primary outcome.

**Table 3:** Multivariate COX regression assessing the association between baseline characters and progression to CKD stage 5.

**Characters**	**Lower stages group** ***N*** **=** **608**		**Higher stages groups** ***N*** **=** **211**	
	**HR (95% CI)**	***P*-value**	**HR (95% CI)**	***P*-value**
Age, years	**1.21** **(1.10, 1.34)**	0.0001	0.98 (0.93, 1.04)	0.4673
Male sex	0.99 (0.48, 2.01)	0.9708	1.09 (0.72, 1.66)	0.6852
Stages of CKD	2.09 (0.92, 4.79)	0.0798	**2.31** **(1.48, 3.62)**	0.0002
Disease				
Nephrotic syndrome	Reference		Reference	
Lupus nephritis	1.03 (0.43, 2.50)	0.9414	0.57 (0.30, 1.08)	0.0851
Immunoglobulin A nephropathy	1.72 (0.56, 5.31)	0.3455	0.67 (0.35, 1.28)	0.2276
Miscellaneous diseases	1.50 (0.54, 4.19)	0.4399	0.86 (0.53, 1.40)	0.5355
Hypertension	1.63 (0.81, 3.26)	0.1690	**1.99** **(1.29, 3.07)**	0.0019
Anemia	1.60 (0.76, 3.39)	0.2174	1.43 (0.94, 2.19)	0.0970
Out-of-pocket payment method	**4.14** **(1.57, 10.95)**	0.0042	0.74 (0.48, 1.15)	0.1796
Medical migration	1.56 (0.74, 3.30)	0.2408	0.85 (0.54, 1.35)	0.4996

*Factors in the multivariate analysis include age, sex, CKD stage, primary disease, hypertension, anemia, payment method, medical migration status*.

*HR, hazard ratio; 95% CI, confidence interval*.

*Bold indicates 95% CI doesn't contain 1.0*.

Sensitivity analysis was carried out by dividing the specific disease as the cause of CKD into two groups: nephrotic syndrome and others in multivariate proportional hazard regression analysis. The results of the sensitivity analyses remained generally consistent with the primary findings. The results of sensitivity analysis were shown in Supplementary Data 6.

## Discussion

A strength of our study is using the big data in HQMS to investigate the association between risk factors and progression of CKD with glomerular etiology in China, an area where the data of CKD in children is scarce. Our study results showed that there is significant heterogeneity of baseline characters between the lower stages group and higher stages group. Further multivariate analysis on each group revealed that risk factors associated with the primary outcome are different. In the lower stages group, older age and out-of-pocket payment methods were found to be associated with the primary outcome. In the higher stages group, CKD stage 4 and hypertension were found to be associated with adverse outcomes.

In lower stages participants, we found that children with out-of-pocket payment methods were associated with a 3.14-times higher risk of progression compared with children with basic medical insurance. However, among the progressors in this group, patients with out-of-pocket payment did not have fewer number of hospitalization compared with those insured patients. How payment method impact on the progression of disease in this group is still unknown, which might be the subject for further studies. A previous study in adults found that lower social-economic status was associated with poor kidney function (29). Children from lower social-economic backgrounds experience reduced quality of life, including less access to medical services compared with their wealthier counterparts (29). In China, basic medical insurance covers up to 70–90% of hospitalized medical costs, thus can greatly reduce economic burdens for families with CKD children. With the support of basic medical insurance, CKD children could have more treatment options in disease management, and better disease management would lead to a better disease prognosis.

Older age was associated with a higher risk of progression in the lower stages group. This result is consistent with the other studies (30). Children with an early diagnosis of CKD may have more opportunities of receiving more attention for disease management, thereby associated with a lower risk of progression. Identifying and diagnosing children with CKD stage 1 or 2 as early as possible might help to reduce the risk of disease progression (31).

In the higher stages group, CKD stage 4 and hypertension were found to be associated with a higher risk of the primary outcome. It's well-established that hypertension is associated with CKD progression in children and adults (6–8, 32). Our results support the previous studies from the childhood CKD population in China.

Even in a relatively short followed-up time, there were 21.0% of the hospitalized children reached the primary outcome. The progression speed is much faster compared to other CKD cohorts studies which mainly consisted of non-glomerular disease and were not restricted to hospitalized patients (5–9, 33). There are two reasons for this difference. Firstly, CKD children with glomerular etiology are at higher risk of progression in previous studies (6, 7, 9, 34). Secondly, compared to patients in the clinic, hospitalized patients tend to be more serious. Thus, hospitalized CKD children with glomerular etiology might have a higher risk for progression. More attention should be paid to this high-risk population. In our study, 7.1% of patients in the lower stages group progressed to stage 5 or dialysis, which has not been reported by other studies yet. This finding suggests that the risk of progression in children with lower stages of CKD cannot be ignored.

The rate of medical migration was 38.2%, which was more than 6 times higher than that of the Chinese adult population (23). This finding reflected the regional disparities of medical accessibility of CKD children in China. Therefore, optimizing the allocation of resources and enhancing the capacity and accessibility of kidney care in vulnerable areas are emerging policy priorities.

This study had several limitations. The first limitation was that the data source was discharge records from a medical quality monitoring system. Only a few clinical and epidemiological parameters were included in this database. Consequently, CKD stages were based on diagnoses only rather than measurement of estimated glomerular filtration rate (eGFR). But we still have proper reasons to applied these classifications according to diagnosis in HQMS in this study. Firstly, discharge diagnoses in HQMS are the official records with legal effect. The diagnosis in HQMS was made by well-trained doctors working in tertiary hospitals and double-checked by their superior doctors according to the current medical management policy to ensure the accuracy. Second, because specific ICD-10 codes were applied to different stages of CKD when extracting the corresponding diagnosis from the system, the results can maintain a certain degree of accuracy. Similarly, because the included database does not include very rich variables, for example, urine protein and specific information of blood pressure were not included, so our results cannot fully explain all possible risk factors and the subgroup analysis of different stages of hypertension could not be achieved. Prospective studies with more comprehensive survey information are still needed to verify our findings. The second limitation in our study is that only hospitalized records were collected in HQMS and some portion of patients with CKD would not be included. Most children with CAKUT, especially in the early stages, have an outpatient follow-up and are therefore not included in the HQMS inpatient database. However, under the current medical system in China, outpatient clinics, in most cases, are not sufficient to make an accurate diagnosis of CKD in children, especially in glomerular etiology. Therefore, it is surmised that CKD patients diagnosed in hospital account for the vast majority of all CKD patients. Nonetheless, more efforts are needed to study all CKD children in China.

## Conclusions

Our study assessed potential risk factors according to different stages of CKD. Age and out-of-pocket payment methods were significantly associated with the primary outcome in childhood CKD stages 1 and 2. Early diagnosis of CKD and increased social medical insurance coverage in early CKD stages, may help to slow down the speed of disease progression. Hypertension was associated with higher risk of the primary outcome in CKD stage 3 and 4. More initiatives should be taken among patients with higher CKD stage and hypertension, to delay the progression process. The rate of medical migration in children is much higher than in adults. Disparities in medical resources between regions should be improved to enhance the accessibility of kidney care for children.

## Data Availability Statement

The datasets presented in this article are not readily available due to the requirement of the Hospital Quality Monitoring System (HQMS) database management rules. Requests to access the datasets should be directed to Jie Ding, djnc_5855@126.com.

## Ethics Statement

The studies involving human participants were reviewed and approved by the Ethics Committee Board of Peking University First Hospital [Approval Number: 2021 (009)]. Written informed consent from the participants' legal guardian/next of kin was not required to participate in this study in accordance with the national legislation and the institutional requirements.

## Author Contributions

Material preparation was performed by GH, HW, YS, and LG. Data collection and analysis were performed by GH, CL, XZ, FW, and JD. The first draft of the manuscript was written by GH, CL, and JD. All authors contributed to the study's conception and design, revised the manuscript critically for important intellectual content, commented on previous versions of the manuscript, and read and approved the final manuscript.

## Conflict of Interest

The authors declare that the research was conducted in the absence of any commercial or financial relationships that could be construed as a potential conflict of interest.

## Publisher's Note

All claims expressed in this article are solely those of the authors and do not necessarily represent those of their affiliated organizations, or those of the publisher, the editors and the reviewers. Any product that may be evaluated in this article, or claim that may be made by its manufacturer, is not guaranteed or endorsed by the publisher.
