# Supplementary material for: Risk Factors for Progression of Chronic Kidney Disease With Glomerular Etiology in Hospitalized Children
Source: Front Pediatr. 2021 Oct 22;9:752717. doi: 10.3389/fped.2021.752717 (PMC8570116; doi:10.3389/fped.2021.752717)
Supplement: Supplementary file 1 [file Table_1.DOCX]

**Supplementary data 1.** Baseline Characteristics of 2442 patients excluded because of the diagnosis of CKD stage 5 or dialysis on the first hospitalization

| **Characteristics** | **Excluded patients**  **N=2442** |
| --- | --- |
| Sex, N (%) |  |
| Male | 1397 (57.2) |
| Female | 1037 (42.5) |
| Unknown | 8 (0.3) |
| Age, years | 14.3 [11.1, 16.5] |
| Etiology, N (%) |  |
| Glomerular disease | 883 (36.2) |
| Non-glomerular disease | 769 (31.5) |
| Unknown | 790 (32.4) |
| Disease, N (%) |  |
| Nephrotic syndrome | 507 (20.8) |
| Lupus nephritis | 137 (5.6) |
| IgA nephropathy | 66 (2.7) |
| Hypertension, N (%) | 911 (37.3) |
| Anemia, N (%) | 1353 (55.4) |
| Payment methods, N (%) |  |
| Out-of-pocket | 1364 (55.9) |
| Basic medical insurance | 764 (31.3) |
| Unknown | 314 (12.9) |
| Medical migration, N (%) |  |
| Yes | 442 (18.1) |
| No | 1919 (78.6) |
| Unknown | 81 (3.3) |

Values for categorical variable are given as number (percentage); value for age as median [Interquartile range].
