# Supplementary material for: Risk Factors for Progression of Chronic Kidney Disease With Glomerular Etiology in Hospitalized Children
Source: Front Pediatr. 2021 Oct 22;9:752717. doi: 10.3389/fped.2021.752717 (PMC8570116; doi:10.3389/fped.2021.752717)
Supplement: Supplementary file 2 [file Table_2.DOCX]

**Supplementary data 2.** Specific diseases/disease groups in 207 patients excluded for nonglomerular etiology

| Disease/Disease group | Number(%) |
| --- | --- |
| Tubular and intestinal disorder | 35 (16.9%) |
| CAKUT^†^ | 34 (16.4%) |
| Metabolic kidney disease | 21 (10.1%) |
| Cystic kidney disease | 17 (8.2%) |
| Obstruction uropathy | 10 (4.8%) |
| Hemolytic uremic syndrome | 8 (3.9%) |
| Infection related kidney disease | 7 (3.4%) |
| Kindey vessel disease | 6 (2.9%) |
| Other known cause | 69 (33.3%) |

^†^CAKUT: Congenital anomalies of the kidney and urinary tract
