# Supplementary material for: Risk Factors for Progression of Chronic Kidney Disease With Glomerular Etiology in Hospitalized Children
Source: Front Pediatr. 2021 Oct 22;9:752717. doi: 10.3389/fped.2021.752717 (PMC8570116; doi:10.3389/fped.2021.752717)
Supplement: Supplementary file 3 [file Table_3.DOCX]

**Supplementary data 3.** Specific diseases/disease groups in the 819 patients enrolled

| Disease/Disease group | Number(%) |
| --- | --- |
| Nephrotic syndrome^†^ | 441 (53.8) |
| Lupus nephritis | 137 (16.7) |
| immunoglobulin A (IgA) nephropathy | 88 (10.7) |
| Henoch-Schönlein purpura nephritis | 78 (9.5) |
| Alport syndrome | 24 (2.9) |
| ANCA glomerulonephritis | 22 (2.7) |
| Membrane nephropathy | 12 (1.5) |
| Lipoprotein nephropathy | 11 (1.3) |
| Other hereditary nephritis | 4 (0.5) |
| immunoglobulin M (IgM) nephropathy | 1 (0.1) |
| WT1 mutation associated nephropathy | 1 (0.1) |

^†^The group of “Nephrotic syndrome” with 441 patients included 93 with pathological diagnosis of focal segmental glomerular sclerosis, 82 with pathological diagnosis of minimal change disease, 21 with other known pathological diagnosis, and 245 without pathological diagnosis.
