# Supplementary material for: Risk Factors for Progression of Chronic Kidney Disease With Glomerular Etiology in Hospitalized Children
Source: Front Pediatr. 2021 Oct 22;9:752717. doi: 10.3389/fped.2021.752717 (PMC8570116; doi:10.3389/fped.2021.752717)
Supplement: Supplementary file 4 [file Table_4.DOCX]

**Supplementary data 4.** Baseline Characteristics of 819 patients classified by outcome

| **Characteristics** | **Progress**  **N=172** | **Nonprogress**  **N=647** | ***P* -value** |
| --- | --- | --- | --- |
| Male sex, N (%) | 105 (61.0) | 393 (60.7) | 0.9420 |
| Age, years | 14.1 [11.5, 16.6] | 11.3 [7.2, 15.0] | <0.0001 |
| Stages of CKD, N (%) |  |  | <0.0001 |
| CKD 1 | 26 (15.1) | 523 (80.8) |  |
| CKD 2 | 17 (9.9) | 42 (6.5) |  |
| CKD 3 | 67 (39.0) | 60 (9.3) |  |
| CKD 4 | 62 (36.0) | 22 (3.4) |  |
| Disease, N (%) |  |  | <0.0001 |
| Nephrotic syndrome | 84 (48.8) | 257 (55.2) |  |
| Lupus nephritis | 28 (16.3) | 109 (16.8) |  |
| IgA nephropathy | 19 (11.0) | 69 (10.7) |  |
| Miscellaneous diseases^†^ | 41 (23.8) | 112 (17.3) |  |
| Hypertension, N (%) | 98 (57.0) | 177(27.4) | <0.0001 |
| Anemia, N (%) | 100 (58.1) | 235(36.3) | <0.0001 |
| Payment methods, N (%) |  |  | 0.0569 |
| Out-of-pocket | 100 (58.1) | 450 (69.6) |  |
| Basic medical insurance | 56 (32.6) | 176 (27.2) |  |
| Unknown | 16 (9.3) | 21 (3.2) |  |
| Total number of hospitalization, N | 816 | 3273 |  |
| Medical migration, N (%) |  |  | 0.2323 |
| Yes | 59 (34.3) | 254 (39.3) |  |
| No | 112 (65.1) | 389 (60.1) |  |
| Unknown | 1 (0.6) | 4 (0.6) |  |

Values for categorical variable are given as number (percentage); value for age as median [Interquartile range].

^†^The group of “Miscellaneous diseases” with 153 patients (41 in nonprogress group and 112 in progress group) included 78 with Henoch-Schönlein purpura nephritis, 24 with Alport syndrome, 22 with ANCA glomerulonephritis, 12 with membrane nephropathy, and 17 with other known diagnosis.
