# Supplementary material for: Risk Factors for Progression of Chronic Kidney Disease With Glomerular Etiology in Hospitalized Children
Source: Front Pediatr. 2021 Oct 22;9:752717. doi: 10.3389/fped.2021.752717 (PMC8570116; doi:10.3389/fped.2021.752717)
Supplement: Supplementary file 5 [file Table_5.DOCX]

**Supplementary data 5.** Baseline Characteristics of 647 patients in nonprogress group classified by CKD stages

| Characteristics | CKD 1  N=523 | CKD 2  N=42 | CKD 3  N=60 | CKD 4  N=22 |
| --- | --- | --- | --- | --- |
| Male sex, N(%) | 15 (57.7) | 10 (58.8) | 46 (68.7) | 34 (54.8) |
| Age, years | 10.5 [6.2，14.4] | 12.1 [8.2，14.7] | 13.8 [11.7，16.7] | 13.2 [9.8，15.9] |
| Disease, N(%) |  |  |  |  |
| Nephrotic syndrome | 312 (59.7) | 18 (42.9) | 22 (36.7) | 5 (22.7) |
| Lupus nephritis | 78 (14.9) | 6 (14.3) | 20 (33.3) | 5 (22.7) |
| IgA nephropathy | 51 (9.8) | 8 (19.0) | 7 (11.7) | 3 (13.6) |
| Miscellaneous diseases | 82 (15.7) | 10 (23.8) | 11 (18.3) | 9 (40.9) |
| Hypertension, N(%) | 135 (25.8) | 11 (26.2) | 23 (38.3) | 8 (36.4) |
| Anemia, N(%) | 169 (32.3) | 17 (40.5) | 34 (56.7) | 15 (68.2) |
| Payment methods, N(%) |  |  |  |  |
| Out-of-pocket | 385 (73.6) | 21 (50.0) | 34 (56.7) | 10 (45.5) |
| Basic medical insurance | 128 (24.5) | 19 (45.2) | 20 (33.3) | 9 (40.9) |
| Unknown | 10 (1.9) | 2 (4.8) | 6 (10.0) | 3 (13.6) |
| Medical migration, N(%) |  |  |  |  |
| Yes | 195 (37.3) | 21 (50.0) | 27 (45.0) | 11 (50.0) |
| No | 324 (62.0) | 21 (50.0) | 33 (55.0) | 11 (50.0) |
| Unknown | 4 (0.8) | 0 (0.0) | 0 (0.0) | 0 (0.0) |

Values for categorical variable are given as number (percentage); value for age as median [Interquartile range].
