# Supplementary material for: Risk Factors for Progression of Chronic Kidney Disease With Glomerular Etiology in Hospitalized Children
Source: Front Pediatr. 2021 Oct 22;9:752717. doi: 10.3389/fped.2021.752717 (PMC8570116; doi:10.3389/fped.2021.752717)
Supplement: Supplementary file 6 [file Table_6.DOCX]

**Supplementary data 6.** COX proportional hazard regression assessing association between baseline characters and CKD progression (specific diseases as cause were classified into nephrotic syndrome and others for sensitivity analysis)

| **Characters** | **Lower stages Group**  **N=608** | | **Higher stages group**  **N=211** | |
| --- | --- | --- | --- | --- |
|  | **HR (95%CI)** | ***P*-value** | **HR (95%CI)** | ***P*-value** |
| Age, years | **1.20 (1.09, 1.33)** | 0.0002 | 0.98 (0.92, 1.04) | 0.4381 |
| Male sex | 1.09 (0.55, 2.14) | 0.8110 | 1.09 (0.74, 1.63) | 0.6601 |
| Stages of CKD | 2.13 (0.93, 4.87) | 0.0748 | **2.52 (1.66, 3.81)** | <0.0001 |
| Nephrotic syndrome | 0.78 (0.38, 1.59) | 0.4937 | 1.39 (0.94, 2.06) | 0.1007 |
| Hypertension | 1.56 (0.79, 3.09) | 0.2003 | **1.85 (1.21, 2.81)** | 0.0042 |
| Anemia | 1.54 (0.73, 3.23) | 0.2543 | 1.34 (0.89, 2.01) | 0.1619 |
| Out-of-pocket payment method | **4.28 (1.62, 11.30)** | 0.0033 | 0.73 (0.48, 1.12) | 0.1526 |
| Medical migration | 1.51 (0.72, 3.16) | 0.2778 | 0.89 (0.57, 1.40) | 0.6248 |

Factors in the multivariate analysis include age, sex, CKD stage, primary disease (nephrotic syndrome or not), hypertension, anemia, payment method, medical migration status.

HR hazard ratio; 95% CI confidence interval.

**Bold** indicates 95% confidence interval doesn’t contain 1.0.
